# A Gesture Recognition Method with a Charge Induction Array of Nine Electrodes

**DOI:** 10.3390/s22031158

**Published:** 2022-02-03

**Authors:** Hao Qian, Yangbin Chi, Zining Dong, Feng Yan, Limin Zhang

**Affiliations:** School of Electronic Science and Engineering, Nanjing University, Nanjing 210023, China; mf1923028@smail.nju.edu.cn (H.Q.); mf20230018@smail.nju.edu.cn (Y.C.); mf1823012@smail.nju.edu.cn (Z.D.); fyan@nju.edu.cn (F.Y.)

**Keywords:** charge induction, gesture recognition, digital input, direction input, key input

## Abstract

In order to develop a non-contact and simple gesture recognition technology, a recognition method with a charge induction array of nine electrodes is proposed. Firstly, the principle of signal acquisition based on charge induction is introduced, and the whole system is given. Secondly, the recognition algorithms, including the pre-processing algorithm and back propagation neural network (BPNN) algorithm, are given to recognize three input modes of hand gestures, digital input, direction input and key input, respectively. Finally, experiments of three input modes of hand gestures are carried out, and the recognition accuracy is 97.2%, 94%, and 100% for digital input, direction input, and key input, respectively. The outstanding characteristic of this method is the real-time recognition of three hand gestures in the distance of 2 cm without the need of wearing any device, as well as being low cost and easy to implement.

## 1. Introduction

With the progress of science and technology, people’s information security is becoming more and more important. However, most applications involving key input or digital input use the contact panel keyboard as the main input device, which will leave fingerprint information, as well as the health problem for public input equipment. Therefore, it is necessary to achieve non-contact hand gesture input, for example, handwriting digital recognition, wave direction recognition, key input recognition, etc.

For handwriting digits recognition, the main methods are to collect the data with the camera and use a digital processing algorithm, such as a neural network algorithm or deep learning algorithm for recognition. For example, the error back propagation neural network is often used for classification and recognition. Various different optimization methods are studied to achieve different recognition accuracy. The neighborhood of the optimal number of hidden neurons of a single hidden layer error back propagation neural network is proposed to ensure convergence without local minima by setting the upper bound as 250 and the lower bound as 5 [1]. Fan et al. introduce a sequence recognition technique adopting a full convolutional sequence recognition network to extract water meters from the images, which could capture context information with less parameters and computation load [2]. A new optical model is given for handwriting digits recognition with the accuracy as high as 93.54% [3]. Guo et al. present a hybrid deep learning model based on a Convolutional Neural Network (CNN) for image recognition, where CNN is responsible for feature extraction, and the model is used as a classifier to complete the final classification, where an error rate of 0.67% is achieved for a specified digit database [4]. Meng et al. put forward three methods to calculate the curvature of gray curve of input image based on curvature coefficient, biquadratic interpolation, and gradient vector interpolation to improve the accuracy of handwritten numeral recognition [5]. Rajashekararadhya et al. invent a hybrid feature extraction algorithm to recognize handwritten digits of two popular South Indian characters, and the recognition rates are 97.75% and 93.9%, respectively [6]. Dhandra et al. propose a probabilistic neural network (PNN) classifier to classify Kanada, Telugu, and Devanagari numbers, and set different radial values for Kannada, Telugu, and Devanagari digital data sets for algorithm verification [7]. Abdulhussain et al. introduce a new scheme for handwritten numeral recognition using hybrid orthogonal polynomials, where the embedded image kernel technique has been adopted in this system, and a support vector machine is used to classify the extracted features for the different numerals. The proposed scheme is evaluated under three different numeral recognition datasets: Roman, Arabic, and Devanagari [8]. Chen et al. introduce an approach for the handwritten digit recognition based on the Saak transform. They use an SVM classifier and the Saak coefficient as classification parameters, and results show that the best one is 98.58% [9]. Alghazo et al. propose a novel structural feature to achieve numeral recognition with several classifiers. The proposed method is tested on six different popular languages, including Arabic Western, Arabic Eastern, Persian, Urdu, Devanagari, and Bangla, and the results of random forest classification can reach 90% [10].

As for non-contact gesture recognition, most of the existing methods collect gesture signals based on video images and use deep learning algorithms for recognition. Kirishima et al. present a comprehensive framework for real-time recognition of unspecified gestures by anyone with the robust ability for various clothing, posture, and motion trajectory [11]. A trajectory classification technology is proposed to realize free gesture recognition and the accuracy can reach 98% [12]. Chen et al. set up a gesture recognition system for continuous gestures recognition under a static background, where the system consists of four modules, real-time gesture tracking and extraction, feature extraction, hidden Markov model training, and gesture recognition, and the recognition accuracy is more than 90% [13]. A multi-dimensional dynamic time warping algorithm for gesture recognition is proposed, where six features are automatically extracted from each pair of frames obtained by two cameras to form a multi-dimensional sequence and find the best synchronization for the recognition [14]. Cheng et al. use the Kinect sensor to obtain the color and depth gesture samples and propose a joint network of CNN and RBM to recognize static gestures [15].

Recently, leapmotion has become more and more popular as a non-contact input method, and some people use it to implement a non-contact PC keyboard [16]. The leap sensor reconstructs the motion information of the palm in the three-dimensional space according to the pictures captured by the built-in two cameras from different angles. The detection range is generally between 25 mm and 600 mm above the sensor, and the detection space is generally an inverted pyramid.

For the above non-contact recognition methods, the system generally requires a camera to collect signals, a high-performance computer to perform algorithm operation and recognize gestures through images and videos at the expense of complexity and high cost.

In addition, there is some research on wearable gesture recognition for non-contact gesture recognition. Moin et al. propose a wearable surface electromyography biosensing system that is based on a screen-printed, conformal electrode array and has in-sensor adaptive learning capabilities. The system can classify gestures in real time, and train and update the model when the environmental conditions change [17]. Mahmoud et al. introduce a system with two EMG channels on the flexor and extensor muscles of the forearm to obtain high-dimensional feature space, where the vector machine (SVM) is selected as a classifier for gesture classification and the accuracy of gesture classification can reach 95% to 100% [18]. However, the user has to wear the specified device for gesture recognition, which brings inconvenience to daily activities.

A non-contact method using infrared sensors is proposed to detect four motion directions, forward, backward, left and right, where a 4 × 5 array of infrared sensors is used as a non-contact input device to detect the change of the motion distance and output corresponding analog voltages [19]. However, the disadvantage of the infrared sensor is that the nonlinearity relation between the output analog voltage and the motion distance, especially for a distance less than 4 cm away, which leads to the difficulty of distinguishing complex gestures, such as digital input.

Amin et al. propose a method of hand gesture recognition based on radar and KNN to classify the micro-Doppler (MD) signal envelope of hand gestures. The experiment with 15 kinds of gestures, such as waving from left to right and waving from right to left, can achieve over a 96% classification rate [20]. A gesture recognition method is proposed based on an ultrasound, where the micro-Doppler effect of fingers is classified by CNN. The proposed method is used to evaluate five finger gestures, including finger close, finger open, finger clockwise circle, finger counterclockwise circle, and finger slide, and the average accuracy of the optimized recognition model is 96.51% [21]. As mentioned above, simple gestures can be recognized by radar and ultrasound, but whether complex gestures such as handwritten digits can be recognized remains to be verified.

Another non-contact method based on charge induction is proposed for hand motion direction recognition by our group, where three electrodes are used to detect the charge variety excited by hand motion, and the direction is calculated by the differences of zero crossing time points of three electrode signals [22]. However, it is difficult to apply the method to recognize the gesture, such as the digital input gesture. 

Therefore, the aim of this paper is to propose a gesture recognition method based on charge induction and the recognition algorithms, including the pre-processing algorithm and back propagation neural network (BPNN) algorithm, which can recognize three input modes of hand gestures, digital input, direction input, and key input. This is the new contribution of this study. Here, a charge induction array of nine electrodes is used to collect gesture signals, as well as simulate a modern intelligent digital dial. Considering the mechanism of charge induction excited by hand motion is introduced in detail in the reference published by our group [22], this paper focuses on system development and algorithm implementation. The paper is organized as follows. Firstly, the principle of signal acquisition based on charge induction is described briefly and the system based on a charge induction array of nine electrodes is given. Secondly, the recognition algorithms, including the pre-processing algorithm and BPNN algorithm, are given to recognize three input modes of hand gestures, digital input, direction input, and key input, respectively. Finally, experiments of three input modes of hand gestures are carried out to verify the feasibility of the proposed method.

## 2. System

When the hand or finger is moving, its body surface will interact with the air, lead to electric charge separation effect, and form a charged body. The induced charge on the electrode can be expressed as

$$Q\left(Q_{0},\;x,\;y,\;z\right)=\left(-\frac{Q_{0}A}{4\pi }\right)\frac{z}{\sqrt{x^{2}+y^{2}+z}{\;}^{3}}$$

where *A* is the area of the electrode, *Q* is the induced charge generated by the charged body $Q_{0}$ on the electrode, and (*x*, *y*, *z*) is a coordinate system with the electrode plane as the *x*–*y* plane and the vertical plane as the *z*-axis. It can be seen that, with the increase of *z*, that is, the farther away from the electrode, the less induced charge is generated. When there are multiple electrodes with the same vertical distance *z*, the farther the electrode is from the fingertip, the greater the calculated value of $x^{2}+y^{2}$, and the smaller the charge generated. In fact, appropriately setting the electrode size and spacing can effectively suppress the interference from adjacent electrodes. When the center distance between two adjacent electrodes is set to 2 cm, the induced charge amplitude ratio of adjacent electrodes is about 2.8:1, which can suppress primary interference from adjacent electrodes. Subsequently, the interference can be removed by the processing algorithm further.

**Figure 1:**
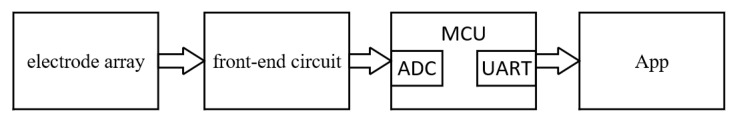
System block diagram.

**Figure 2:**
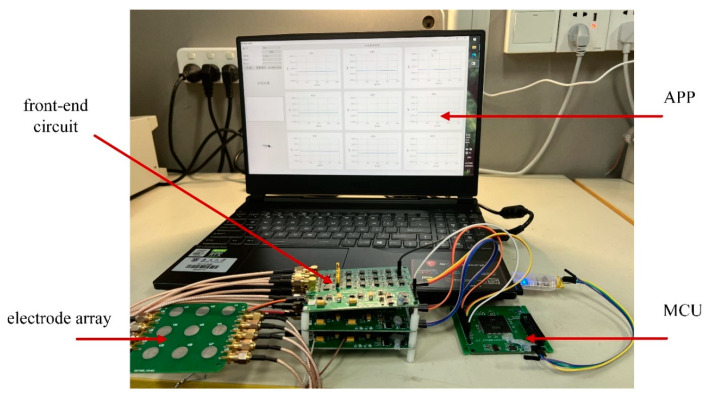
System device.

The system block diagram is shown in Figure 1 and the system device is shown in Figure 2, including the hardware acquisition circuit and the application (APP) in the computer, where the hardware acquisition system is composed of electrode array, front-end circuit, and microcontroller (MCU). The microcontroller transmits the gesture signals collected by an electrode array and front-end circuit to the APP through a universal asynchronous receiver/transmitter (UART). The APP realizes gesture recognition, displays the collected signals, and provides man–machine interface. The system can realize three input modes of hand gestures, digital input, direction input and key input, respectively. For the digital input mode, the user can write numbers with one finger over the electrode array about 2 cm. For the direction input mode, the user can slide up, down, left, and right with one finger over the electrode array about 2 cm. For the key input mode, the user can approach the key with one finger over the electrode array from far to near. 

### 2.1. Hardware Acquisition Circuit

**Figure 3:**
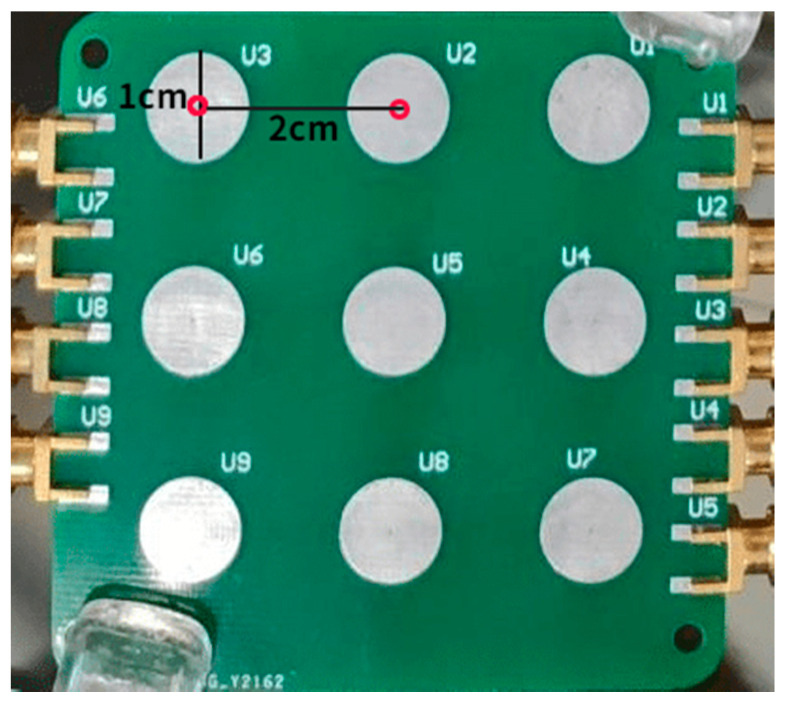
Induction electrode array.

As described above, the hardware acquisition system includes three parts: electrode array, front-end circuit, and microcontroller, which are used to collect gesture signals and upload the signal data to the APP through the UART of the microcontroller. The electrode array is shown in Figure 3, where nine electrodes are arranged according to 3 × 3 matrix arrangement, and each electrode is a circular electrode with a diameter of 1 cm and tin plating on its surface. The center distance between two adjacent electrodes is set to 2 cm to ensure that, when the distance of the finger from the electrode plane is about 2 cm, the signal amplitude ratio collected by the adjacent electrodes can exceed 2.8:1.

**Figure 4:**
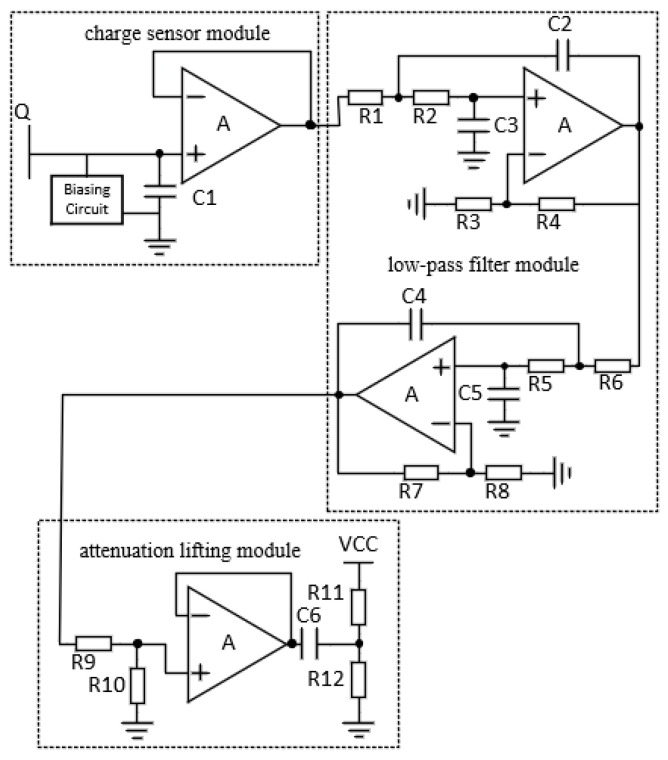
Schematic diagram of front-end circuit.

The front-end circuit is used to collect the charge induced by the electrode array, convert the charge to the voltage, and filter the signal further to output to the analog digital convertor (ADC) of the microcontroller. The schematic diagram of the front-end circuit is shown in Figure 4, including charge sensor module, low-pass filter module, differential circuit module and attenuation lifting module. The charge sensor module converts the charge sensed by the electrode into voltage signal through the charge sensing chip, where the equivalent input capacitance is about 10 pF. The low-pass filter module is a fourth-order low-pass filter with a cut-off frequency of 8 Hz and a gain of 10, where the cut-off frequency is set according to the low frequency characteristic of the charge signal excited by the gesture. The attenuation lifting module realizes the signal with 1/2 attenuation and DC bias of 1/2 reference voltage of the ADC in order to meet the ADC input range. The main functions of microcontroller are analog-to-digital conversion and data transmission through UART port to the APP. In this implementation, the chip STM32F103 is selected as the microcontroller, its built-in 12-bit ADC is used to realize the nine-channel signal analog-to-digital conversion function.

### 2.2. The Application

**Figure 5:**
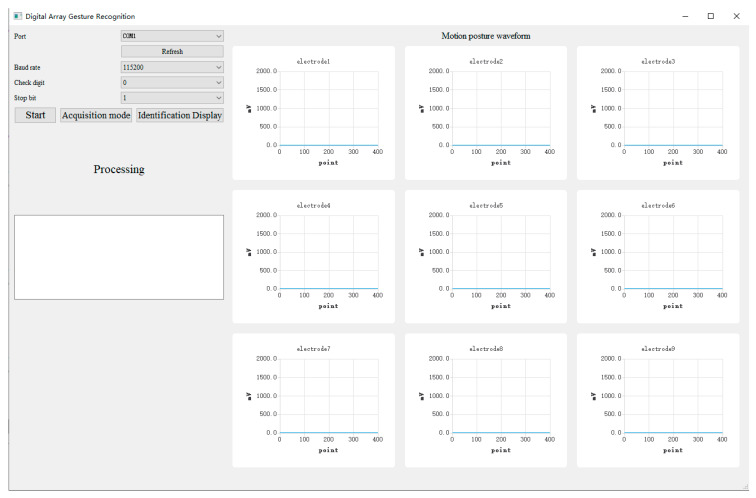
Main window of the application.

**Figure 6:**
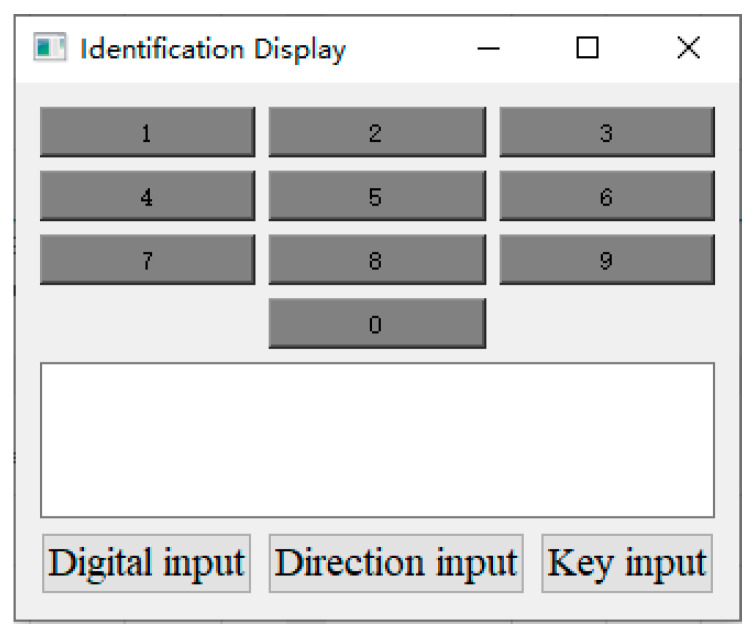
Chip window of the application.

The function of the APP is to display the collected signals from the hardware acquisition circuit, provide man–machine interface, and realize gesture recognition. The APP is developed with Qt software, which is divided into two windows: main window and chip window. As shown in Figure 5, the main window is used to display and save signals transmitted from hardware acquisition circuit, where the control area on the left side of the main window is used to set serial port number, baud rate and other information to connect with a hardware acquisition circuit, and provide the man–machine interaction, including “Start”, “Acquisition mode”, and “Identification Display” further. The “Start” button is used to start the system. The “Acquisition mode” button is used to collect data with a 4 s interval and save them to the local disk, and the collection status will be shown in the “Processing” textbox. The “Identification Display “button is used to call a chip window to display the recognition results. As shown in Figure 6, the chip window can be used to select the input mode and display the recognition results. For the key input, the “Key input” button is highlighted in red, and the corresponding input key is highlighted in green. For the direction input, the “Direction input” button is highlighted in red, and the keyboard array can dynamically display the recognition direction. For example, when the identification direction is “up”, the keyboard array will be highlighted dynamically in green from 0 to 2 at an interval of 1 s, indicating the current direction, and the result will be further explained in the text box. For the digital input, the “Digital input” button is highlighted in red, the handwriting numbers 0–9 are identified, and the recognition results are displayed in the text box.

In order to solve the time-consuming of recognition algorithm and display the collected signals and recognition results in real time, the APP is designed with multi-thread architecture. There are three threads, where the main thread is used to display and save signals from hardware acquisition circuit and send them to the python thread, the chip thread is designed to select the input mode and display the recognition result and the python thread is applied to recognize the input mode with the proposed recognition algorithm and send the recognition results to the chip thread.

## 3. Recognition Algorithm

**Figure 7:**
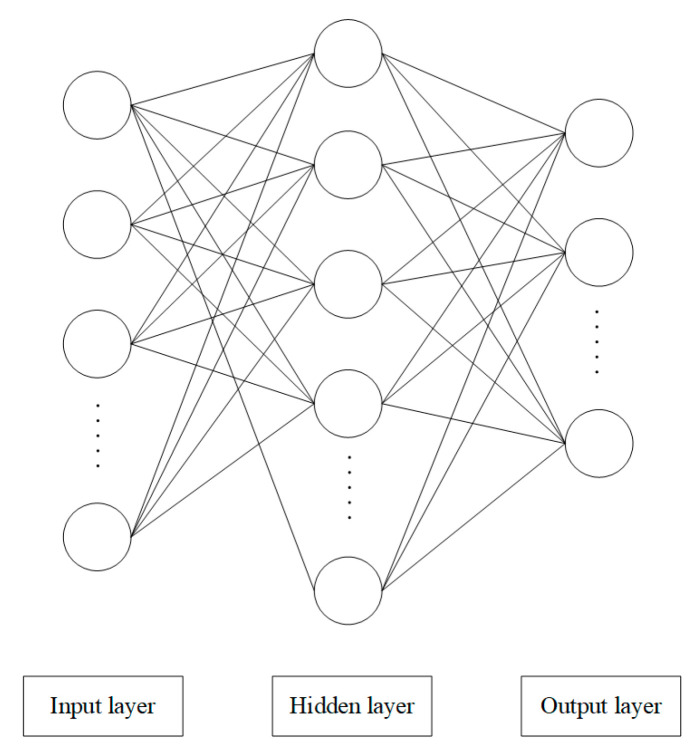
BPNN structure diagram.

The recognition algorithm is mainly based on the classification of the signal characteristics. For the digital input, the signals generated by handwritten digits 0–9 are classified. For the direction input, the signals generated in the up, down, left, and right directions are classified. To simplify the classification, some pre-processing algorithms are used. Here, the widely used BPNN algorithm is adopted for classification training and recognition [23,24,25], and the BPNN structure is shown in Figure 7. The nodes of the BP method are divided into three layers, input layer, hidden layer, and output layer. The dimension of the input data can be set according to the application requirements. The number of the hidden layer can be adjusted according to the classification accuracy in the training process under the condition greater than or equal to the input layer node, and the initial number of the hidden layer can be set to 1. The output dimension is generally the number of categories classified. At the beginning of training, the appropriate number of iterations and learning rate should be set, for example, 0.01 and 10,000, respectively. The activation function of the algorithm is the tanh function, which can improve the speed of convergence for its zero-centered output feature.

### 3.1. Digital Input Recognition

**Figure 8:**
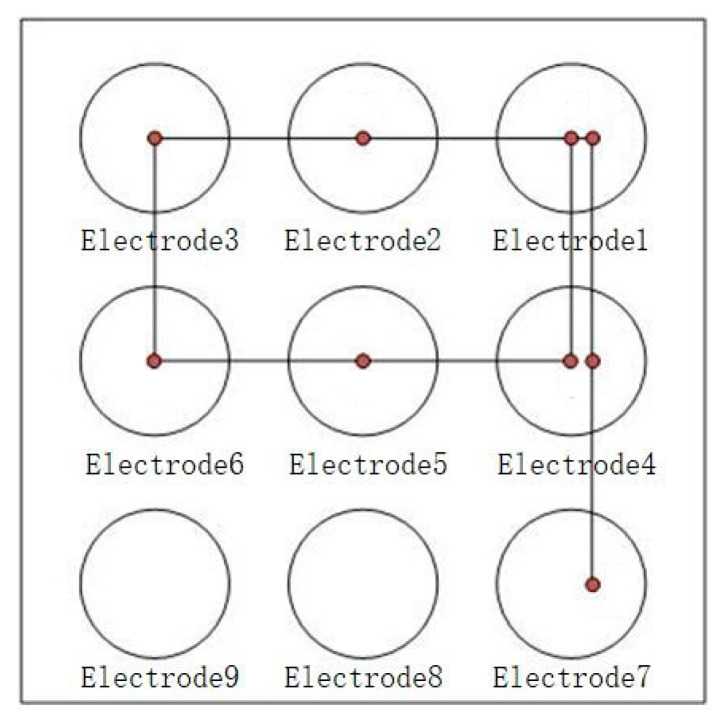
Finger motion path of input digit 9.

**Figure 9:**
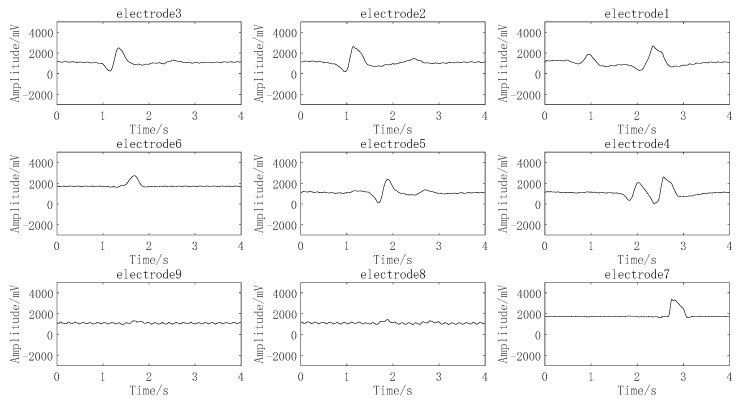
Signals generated by input digit 9.

Digital input is to write any digit of 0–9 with one finger over the electrode array about 2 cm. For example, when writing the digit 9, the finger passes through electrodes 1, 2, 3, 4, 5, 6, 7, 6, and 9 in turn, the motion path is shown in Figure 8, and the signals of corresponding electrode are shown in Figure 9. Other digits are input in the same way. Therefore, based on the motion path, digits can be classified preliminarily by the electrode number of the finger passed, and the digital pattern classification of passed electrode number for every digit are listed in Table 1.

From the results of Table 1, it can be seen that the digits 1, 7, 4, and 0 can be preliminarily identified by the electrode number of the finger passed. Although the digits 9 and 8 have the same passed electrode number, they can be distinguished by the signal of unpassed electrode 4, 6, 7, 8 further. The digits 2, 3, 5, and 6 are all characterized with the nine effective electrodes, and it is necessary to use a BPNN neural network algorithm to recognize them further.

**Figure 10:**
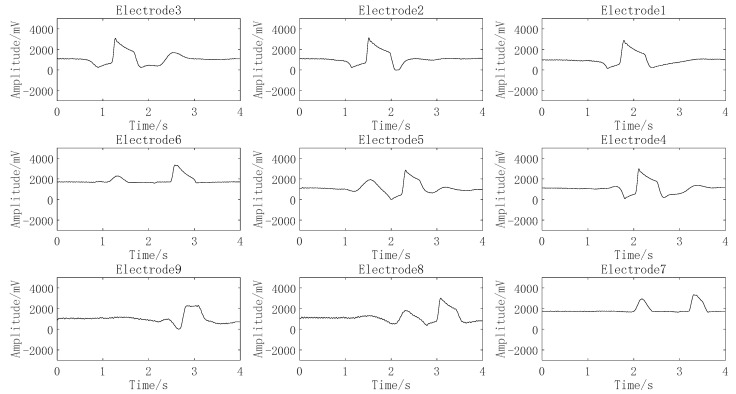
Signals generated by digit 2.

To simplify the network algorithm and improve the recognition accuracy, the signal characteristics of digits 2, 3, 5, and 6 are used for the input of the BPNN neural network. The signal of each electrode generated by input digit 2 is shown in Figure 10, where the signal generated by the finger across the electrode is characterized with one peak or valley, and the generation time point of the signal of each electrode is different and can be sequenced as electrode 3, electrode 2, electrode 1, electrode 4, electrode 5, electrode 6, electrode 9, electrode 8, and electrode 7. Similarly, digit inputs 3, 5, and 6 can lead to different electrode sequences. The electrode sequence corresponding to the signal sequence of each electrode generated by digits 2, 3, 5, and 6 are listed in Table 2, which means the generation time point of effective signal of each electrode can be extracted and used as the input of the BPNN neural network. 

According to a number of experiments, it is found that the peak amplitude of effective signal generated by the digital input is almost concentrated about 450 mV over the DC bias of 1/2 reference voltage of the ADC. Therefore, the generation time point of effective signal can be set as the time that the signal enters in the amplitude range for the first time. Detailed parameters of the BPNN neural network are set as follows: the input node is the generation time point of each electrode and the number of input nodes is 9. The hidden layer should be greater than or equal to the input layer node and is set as 10 here. The output layer is responsible to classify the digits 2, 3, 5, and 6, and the node number is set as 4. The learning rate is 0.01 and the number of iterations is 10,000.

### 3.2. Direction Input Recognition

**Figure 11:**
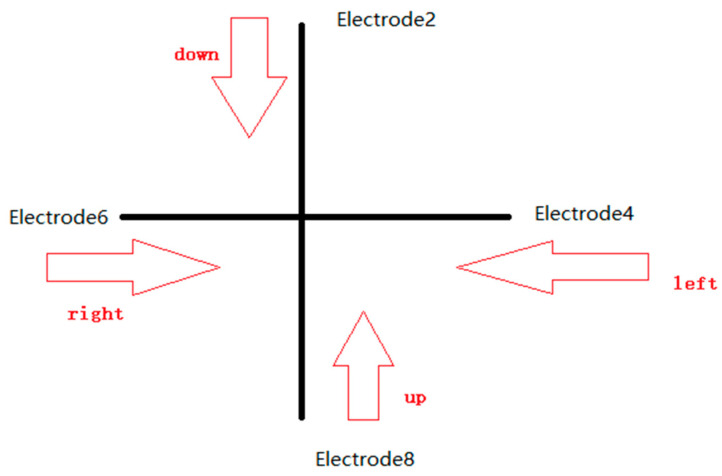
Schematic diagram of direction input.

For direction input, the schematic diagram for four direction is given in Figure 11, where the up direction is the motion of the finger from electrode 8 to electrode 2, the down direction is the motion of the finger from electrode 2 to electrode 8, the left direction is the motion of the finger from electrode 4 to electrode 6, and the right direction is the motion of the finger from electrode 6 to electrode 4. This means that the direction can be recognized by the electrode sequence. Moreover, it is pointed out in Section 3.1 that the signal generated by the finger across the electrode is characterized with one peak or valley and the amplitude about 450 mV over the DC bias of 1/2 reference voltage of the ADC, which can be seen as an effective signal. Therefore, two features are used as the direction recognition, one is the effective signal and the other is the electrode sequence, which are combined as one feature pair. Table 3 shows the detailed feature pairs for four directions, where the first feature is set as 1 for the effective signal and the second feature is set as the electrode sequence for the effective signal and two features are both set as 0 for noneffective signals. 

For example, for the left direction across electrodes 4 and 6 in turn, the first feature of electrode 4 is set 1, the second feature of electrode 4 is set 1, the first feature of electrode 6 is set 1, the second feature of electrode 6 is set 2, and the two features of electrode 2 and 8 are all set 0 for there are no effective signals from them. 

The feature pairs are used as the direction recognition to reduce the complexity of the method, and the recognition steps are given as follows:

(1) According to the first feature of electrodes 2 and 8 to judge the horizontal direction or the vertical direction, where the horizontal direction contains the right direction and left direction and the vertical direction involves the up direction and down direction.
(2) If it is a horizontal direction, the specific direction is determined according to the second feature of electrode 4 and electrode 6. Similarly, if it is a vertical direction, the specific direction is determined according to the second characteristics of electrodes 2 and 8.
(3) After judgment, set the variable flag1 as x in (0,1,2,3), where 0, 1, 2, 3 are the representation of direction left, right, up, and down, respectively.
(4) Then, set the second feature of Table 3 as the input node data of the BPNN neural network. At the same time, set the learning rate as 0.01 and the number of iterations as 10,000.
(5) Similarly, the assignment variable flag2 is set as x in (0,1,2,3) according to the result calculated by BPNN.
(6) Compare the results of flag1 and flag2. If they are the same, output the recognition direction. If they are different, output “this signal is invalid”.

The direction algorithm is a double identification method by comparing two results to improve the robustness of recognition, where, for the first identification, the results are obtained directly through the effective signal and the electrode sequence, and for the second identification, the results are calculated by inputting the second feature in Table 3 into the BPNN algorithm.

### 3.3. Key Input Recognition

For key input, when the finger approaches the electrode, the amplitude of the target electrode will be greater than that of other electrodes. The recognition algorithm is designed based on this phenomenon. Firstly, obtain the peak-to-peak amplitude of the signal of each electrode. Then, find the maximum signal from the nine-electrodes array. Finally, the electrode corresponding to the maximum signal is the input electrode. 

## 4. Experimental Results

In order to verify the effectiveness of the proposed recognition algorithm, experimental verification is carried out. The experiment data are collected by one finger about 2 cm above the electrode array performing digit input, direction input, which are from five people at different times under the condition of room temperature and 62% relative humidity. Two steps are contained in the experiments: one is training the input gesture, and the other one is recognizing the input gesture. The training algorithm is the same as the recognition algorithm, as well as the parameters set. For effective recognition, the digital pattern training set is composed of 970 groups, about 100 groups for each digit, and the directional pattern training set is 350 groups, about 90 groups for each direction.

For digital input, digits 0, 1, 4, 7, 8, and 9 can be classified by the passed electrode with the pre-processing algorithm and digits 2, 3, 5, and 6 are recognized with a BPNN algorithm. For the four digits, all are characterized with nine effective electrodes, and the number of input nodes of BPNN is 9. The hidden layer is similar to the input node, and it is set to 10. The number of output nodes should be the same as the classification number, and it is defined as 4. The learning rate is 0.01, and the iteration number is 10,000.

For direction input, four directions are distinguished by the signal feature of electrodes 2, 4, 6, and 8 with the time sequence and BPNN algorithm for double identification. The second feature representing the electrode sequence shown in Table 3 is used as the input of BPNN algorithm. Therefore, the number of input nodes of BPNN is 4. The hidden layer is similar to the input node, and it is set to 5. The number of output nodes is the same as the classification number, and it is defined as 4. The learning rate is 0.01, and the iteration number is 10,000.

The above algorithms are running on a computer with Intel’s i7-8700 CPU, 8 GB system memory, and a 64-bit windows operating system. After the training, the weight file of the corresponding input mode is obtained to further recognize the test set of the input gesture in real time.

### 4.1. Recognition Accuracy of Digital Input

The test set consists of 500 groups of real-time handwritten digital signals. Each digit has 50 groups of data. The average recognition time is about 500 ms. The recognition results are listed in Table 4, and the overall accuracy is 97.2%. 

### 4.2. Recognition Accuracy of Direction Input

The test set consists of 200 groups of real-time direction signals, about 50 groups for each direction. The average recognition time is less than 500 ms. The recognition results are listed in Table 5, and the overall accuracy is 94%.

### 4.3. Recognition Accuracy of Key Mode

A total of 450 groups of key signals are tested with 50 groups for each key. The recognition time is less than 100 ms. The recognition results are listed in Table 6, and the accuracy is 100%.

## 5. Conclusions

In this paper, a non-contact and simple gesture recognition method is proposed based on charge sensing and a BPNN neural network algorithm. Firstly, the principle of signal acquisition based on charge induction is introduced, and the whole system with a charge induction array of nine electrodes is described. Secondly, the recognition algorithms, including the pre-processing algorithm and back propagation neural network (BPNN) algorithm, are given to recognize three input modes of hand gestures, digital input, direction input, and key input, respectively. Here, different pre-processing methods are proposed for digital input and direction input, which can greatly reduce the computation complexity of the neural network and ensure the real-time recognition. Finally, experiments of three input modes of hand gestures are carried out, and the recognition accuracies are 97.2%, 94%, and 100% for digital input, direction input, and key input, respectively. The outstanding characteristic of this method is the real-time recognition of three hand gestures in the distance of 2 cm without the need for wearing any device, as well as being low cost and easy to implement. In addition, the proposed method without the pre-processing algorithm can be applied to recognize other gestures by adding a new classification label to the neural network algorithm and retraining the added new gesture with adjusted parameters for recognition requirements.

## Figures and Tables

**Table 1:** Digital pattern classification.

Number of Electrodes	Digit
3 electrodes	1
5 electrodes	7
6 electrodes	4
7 electrodes	9, 8
8 electrodes	0
9 electrodes	2 3 5 6

**Table 2:** Time sequence of signals generated by digits 2, 3, 5, and 6.

	Electrode	1	2	3	4	5	6	7	8	9
Digit										
2		3	2	1	4	5	6	9	8	7
3		3	2	1	4	5	6	7	8	9
5		9	8	1	4	3	2	5	6	7
6		1	2	3	8	9	4	7	6	5

**Table 3:** Feature pairs of direction input.

(with or without Peak, Serial Number)	2	4	6	8
Left	(0,0)	(1,1)	(1,2)	(0,0)
right	(0,0)	(1,2)	(1,1)	(0,0)
up	(1,2)	(0,0)	(0,0)	(1,1)
down	(1,1)	(0,0)	(0,0)	(1,2)

**Table 4:** Recognition results of digital input.

Digit	Correct Number	Number of Errors	Total	Correct Rate
0	50	0	50	100%
1	50	0	50	100%
2	46	4	50	92%
3	47	3	50	94%
4	50	0	50	100%
5	46	4	50	92%
6	47	3	50	94%
7	50	0	50	100%
8	50	0	50	100%
9	50	0	50	100%
total	486	14	500	97.2%

**Table 5:** Recognition results of direction input.

Direction	Correct Number	Number of Errors	Total	Correct Rate
Left	50	0	50	100%
Right	46	4	50	92%
Up	45	5	50	90%
Down	47	3	50	94%
total	188	12	200	94%

**Table 6:** Recognition results of key input.

Key	Correct Number	Number of Errors	Total	Correct Rate
Key 1	50	0	50	100%
Key 2	50	0	50	100%
Key 3	50	0	50	100%
Key 4	50	0	50	100%
Key 5	50	0	50	100%
Key 6	50	0	50	100%
Key 7	50	0	50	100%
Key 8	50	0	50	100%
Key 9	50	0	50	100%
total	450	0	450	100%
